# Osteogenic Differentiation of Human Amniotic Fluid Mesenchymal Stem Cells Is Determined by Epigenetic Changes

**DOI:** 10.1155/2016/6465307

**Published:** 2016-10-12

**Authors:** Monika Glemžaitė, Rūta Navakauskienė

**Affiliations:** Department of Molecular Cell Biology, Vilnius University, LT-10257 Vilnius, Lithuania

## Abstract

Osteogenic differentiation of human amniotic fluid derived mesenchymal stem cells (AF-MSCs) has been widely studied* in vitro* and* in vivo* as a potential tool for regenerative medicine and tissue engineering. While most of the studies analyze changes in transcriptional profile during differentiation to date there is not much information regarding epigenetic changes in AF-MSCs during differentiation. The aim of our study was to evaluate epigenetic changes during osteogenic differentiation of AF-MS cells. Isolated AF-MSCs were characterized morphologically and osteogenic differentiation was confirmed by cell staining and determining expression of alkaline phosphatase and osteopontin by RT-qPCR. Variation in gene expression levels of pluripotency markers and specific microRNAs were also evaluated. Analysis of epigenetic changes revealed that levels of chromatin modifying enzymes such as Polycomb repressive complex 2 (PRC2) proteins (EZH2 and SUZ12), DNMT1, HDAC1, and HDAC2 were reduced after osteogenic differentiation of AF-MSCs. We demonstrated that the level of specific histone markers keeping active state of chromatin (H3K4me3, H3K9Ac, and others) increased and markers of repressed state of chromatin (H3K27me3) decreased. Our results show that osteogenic differentiation of AF-MSCs is conducted by various epigenetic alterations resulting in global chromatin remodeling and provide insights for further epigenetic investigations in human AF-MSCs.

## 1. Introduction

Human amniotic fluid derived mesenchymal stem cells (AF-MSCs) are a new stem cell source for regenerative medicine and therapy. AF-MSCs are obtained by amniocentesis and analyzed for prenatal diagnostics of various foetal abnormalities and genetic diseases. Amniotic fluid is known to contain multiple cell types derived from the developing foetus and extraembryonic tissues including foetal skin, placenta membranes, epithelial, and mucosa of foetal digestive, respiratory, and urinary tract [1, 2]. It has been shown that, among other cells that are obtained with the amniocentesis sample, there is a fraction of cells exhibiting stem cell like properties [3]. These cells were termed amniotic fluid derived mesenchymal stem cells because they showed characteristics of mesenchymal stem cells being able to proliferate highly, self-renew, and have multiple lineage differentiation potential towards osteogenic, adipogenic, myogenic, neurogenic, endothelial, and hepatic phenotypes* in vitro* and they even performed better than adult stem cells [4–6]. On the other hand, mesenchymal stem cells derived from amniotic fluid do not support initiation of cancer. AF-MSCs can be obtained from amniocentesis samples securely, avoiding ethical issues related to embryonic stem (ES) cells [5, 7].

Human amniotic fluid derived stem cells express Oct4, Sox2, Nanog, Rex1, and cyclin A as well as mesenchymal stem cell surface markers that include CD90, CD105, CD73, CD166, CD133, and CD44 [3, 8–10]. In addition to this, it was established that AF-MSCs are negative for markers of hematopoietic lineage (CD45) and hematopoietic stem cells (CD133, CD34), confirming the lack of contamination with other cells from the umbilical cord and foetal blood [11].

As mentioned earlier, AF-MSCs have multilineage potential and express pluripotency markers. Considering these properties they are classified as multipotent stem cells sharing characteristics of both embryonic and adult stem cells. AFS cells show no evident antigenicity and therefore can be employed as a tool for a basic research and studied ahead of their use for cell-based therapies [1, 12–14]. Moreover, induced pluripotent stem cells (iPSCs) were generated from AF-MSCs using four Yamanaka factors, OCT4, SOX2, KLF4, and c-MYC [15, 16], two-factor (OCT4 and SOX2) [17] reprogramming system without the use of oncogenes, or even ectopic expression of the only one transcription factor OCT4 [18].

Osteogenic differentiation induction in AF derived mesenchymal stem cells obtained from various sources (human, sheep, mouse, and rat) has been described [10, 19, 20]. It is documented that culturing of AF-MSCs with various agents such as Simvastatin [21], herbal medicines [22, 23], and phytoestrogens [24] or with dental pulp stem cells [25] or specific microRNAs [26] increase osteogenic differentiation. Studies describing the possibilities of* in vivo* osteogenic differentiation of AF derived cells were presented [27, 28]. While most of the studies analyze changes in transcriptional profile during differentiation, epigenetic processes are the other key factors that constitute a molecular basis for transcriptional potential.

Epigenetic factors such as DNA methylation [29, 30] and histone methylation/acetylation together with Polycomb repressive complexes 1 and 2 (PRC1 and PRC2) are identified as main regulators of pluripotency in parallel with Oct4/Nanog in embryonic stem cells. They are also responsible for maintenance of bivalent chromatin structure of developmental genes [31, 32]. Histone modifying enzymes associated with multilineage differentiation of adult mesenchymal stem cells have been reported [33, 34] but to date there is not much information regarding epigenetic changes in amniotic fluid mesenchymal stem cells during differentiation.

In the present study we demonstrated that gene expression level of pluripotency markers (Sox2 and Rex1), the expression of specific microRNAs, chromatin modifying enzymes (EZH2, SUZ12, DNMT1, HDAC1, and HDAC2), and histone modifications (H3K4me3, H3K9Ac, H4 hyperAc, and H3K27me3) were altered after osteogenic differentiation induction in amniotic fluid derived stem cells. The results revealed downregulation of proteins involved in silencing lineage-specific genes and maintaining bivalent state in stem cells as well as enzymes promoting chromatin compaction and changes in histone modifications proposing that osteogenic differentiation is conducted by various epigenetic changes resulting in global chromatin remodeling.

## 2. Materials and Methods

### 2.1. Isolation and Expansion of Mesenchymal Stem Cells from Human Amniotic Fluid

Samples were obtained by amniocentesis from second trimester amniotic fluid from healthy women who needed prenatal diagnostics but no foetus abnormalities were detected by genetic analysis (protocols approved by the Ethics Committee of Biomedical Researches of Vilnius District, number 158200-123-428-122). Amniotic cells were isolated using two-stage protocol as described by Savickienė and coauthors [6] without c-Kit specific antibodies selection. A morphologically homogeneous population of AF-MSCs was maintained in growth medium and subcultured into higher passages at approximately 80% confluence with 0.05% trypsin-EDTA (Gibco, Life Technologies, Grand Island, NY, USA).

### 2.2. Flow Cytometry Analysis

For phenotypical identification of AF-MSCs, at least 1 × 10^5^ cells for one assay were collected by centrifugation at 600 ×g for 5 min. Pelleted cells were washed twice in phosphate-buffered saline (PBS) supplemented with 0.2% foetal bovine serum (FBS) (Gibco, Life Technologies, Grand Island, NY, USA). Then cells were suspended in 50 *μ*L PBS with 1% bovine serum albumin (BSA) and incubated with the following antibodies against cell surface markers: FITC conjugated mouse anti-human CD45 (BD Pharmingen, San Jose, CA, USA), CD34 (Miltenyi Biotec, Teterow, Germany), and CD90 (Molecular Probes, Life Technologies, Waltham, MA, USA) or PE labeled mouse anti-human CD105 (Invitrogen, Life Technologies, Waltham, MA, USA). Mouse IgG2A-FITC (Miltenyi Biotec, Teterow, Germany), IgG1-FITC (Invitrogen, Life Technologies, Waltham, MA, USA), or IgG1-PE (Molecular Probes, Life Technologies, Waltham, MA, USA) was used as isotype controls. Samples were incubated in the dark at 4°C for 30 min and washed twice with PBS with 1% BSA. Finally, cells were suspended in 200 *μ*L PBS with 1% BSA and analyzed using Guava easyCyte 8HT flow cytometer (Millipore, USA) with InCyte 2.2.2 software.

### 2.3. Differentiation Assay

Osteogenic differentiation of AF-MSCs was performed in a monolayer using StemPro® Osteogenesis Differentiation kit (Gibco, Grand Island, NY, USA). AF-MSCs were cultured at 80%–90% confluence and subsequently differentiated with differentiation medium at 37°C in 5% CO_2_. For cell staining, AF-MSCs were seeded into a 4-well (3.85 cm^2^) plate (Nunc, Thermo Fisher Scientific, Roskilde, Denmark) at a 1 × 10^4^ cells/cm^2^ density. Each cell population was differentiated in 3 replicates using undifferentiated cells as a control. Three independent differentiation experiments were performed on separate days. During cell differentiation, the medium was replaced every 2-3 days. After 15 days of differentiation cells were stained as described in [6].

### 2.4. RNA Isolation and RT-qPCR

Total RNA from undifferentiated and differentiated amniotic fluid stem cells was isolated using TRIzol® reagent (Ambion, Carlsbad, CA, Life Technologies).

For gene expression analysis, cDNA was made using Maxima First Strand cDNA Synthesis Kit for RT-qPCR (Thermo Scientific, Vilnius, Lithuania) and Maxima SYBR Green qPCR Master Mix (Thermo Scientific, Vilnius, Lithuania) on the Rotor-Gene 6000 system (Corbett Life Science) was applied. The amount of mRNA was normalized to GAPDH and relative gene expression was calculated using ΔΔ*C*_*t*_ method (compared to undifferentiated control). The primers in Table 1 were used in gene expression analysis.

For microRNA expression analysis, RNA was reverse transcribed into specific microRNA cDNA using Taqman® MicroRNA Reverse Transcription Kit and Taqman MicroRNA Assay (Life Technologies, Foster City, CA, USA). The following microRNA assays were used: hsa-miR-223-3p, hsa-miR-21-3p, hsa-miR-34a-3p, hsa-miR-17-3p, and hsa-miR-148b-3p. MicroRNA expression levels were analyzed using Taqman MicroRNA Assay and Taqman Universal PCR Master Mix II, without UNG (Applied Biosystems, Life Technologies, Foster City, CA, USA). The microRNA levels were normalized to endogenous control RNU48. The relative expression of microRNA was determined using ΔΔ*C*_*t*_ method (compared to undifferentiated control).

### 2.5. Total Protein Isolation and Western Blot Analysis

For total protein extraction differentiated and undifferentiated cells were trypsinized, washed twice with ice-cold PBS, and incubated with benzonase (1/10 volume of cell pellets) (Merck, Darmstadt, Germany) for 30 min on ice. Then cells were resuspended in equal volume of 2x SDS lysis buffer (125 mM Tris, pH 6.8, 4% SDS, 200 mM DTT, 20% glycerol, and traces of bromphenol blue) and 10 volumes of 1x SDS lysis buffer were added. Samples were homogenized through the 26 G needle and heated at 96°C for 5 min. After centrifugation at maximum speed for 15 min at 4°C, the supernatant was collected and used for loading into 7.5–15% gradient polyacrylamide gels. After Tris-glycine SDS electrophoresis, proteins were transferred onto PVDF membrane. Immunoblotting was performed using antibodies against DNMT1, HDAC1, HDAC2 (Santa Cruz Biotechnology, Dallas, Texas, USA), SUZ12, EZH2 (Cell Signaling Technology, Beverly, MA, USA), H3K4me3, H3K27me3, H4 hyperAc (Penta), and H3K9Ac (Millipore, Temecula, CA, USA). Antibodies against GAPDH (Abcam, Cambridge, UK) were used as a protein loading control. Secondary horseradish peroxidase-conjugated anti-mouse, anti-rabbit, and anti-goat antibodies (DAKO, Glostrup, Denmark) were used and enhanced chemiluminescence detection was performed using Super Signal™ West Pico Chemiluminescent Substrate (Thermo Scientific, Rockford, IL, USA) and ChemiDoc™ XRS+ system with Image Lab™ Software (Bio-Rad Laboratories). Semiquantitative analysis of blots was done using ImageJ software (NIH, USA).

### 2.6. Statistical Analysis

Unless otherwise specified, all experiments were repeated at least three times. Data were expressed as mean values with SDs. For statistical analysis two-sample Student's* t*-test was performed and significance was set at ^*∗*^ *P* ≤ 0.05, ^*∗∗*^ *P* ≤ 0.001, and ^*∗∗∗*^ *P* ≤ 0.0001.

## 3. Results

### 3.1. Characterization of AF-MSCs

**Figure 1:**
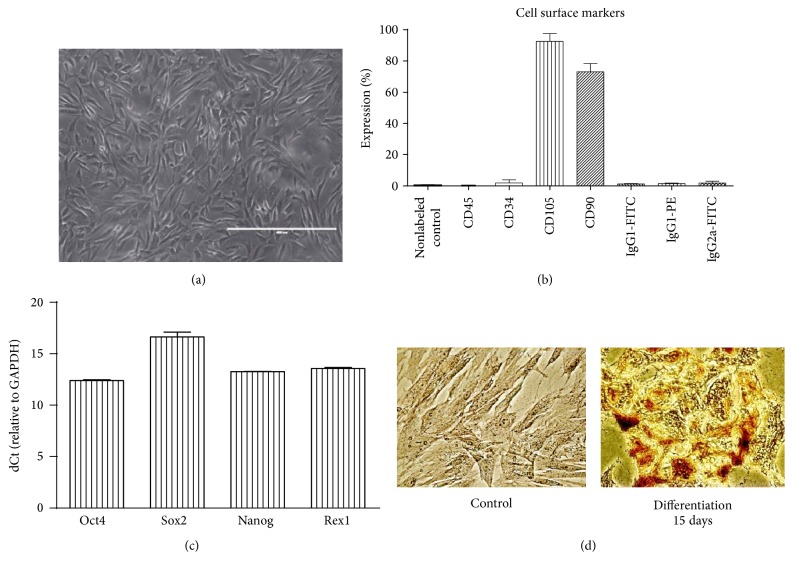
Characterization of AF-MSCs. (a) Representative image of spindle-shaped AF-MSCs. Scale bar = 400 *μ*m. (b) Expression of cell surface markers CD45, CD34, CD105, and CD90 measured by using FACS and FITC or PE labeled antibodies. The data was represented as mean with standard deviation (*n* = 3). (c) Expression of stem cells pluripotency markers* Oct4*,* Sox2*,* Nanog*, and* Rex1* as determined by RT-qPCR. The data were normalized to GAPDH and presented as mean ± SD (*n* = 3). (d) AF-MSCs after induction of osteogenic differentiation for 15 days, stained with Alizarin Red. Calcified extracellular matrix deposition is colored red proving osteogenic differentiation.

Amniotic fluid derived mesenchymal stem cells were obtained from second trimester amniocentesis samples and cultivated using two-stage protocol as described in Section 2. Typical spindle-shaped cells (Figure 1(a)) were used for the following experiments. Using flow cytometry, these cells were characterized as strongly positive for mesenchymal cell surface markers CD105 (endoglin, expression over 90%) and CD90 (Thy-1, thymocyte antigen-1, expression of more than 70% of cells) and negative for CD34 (hematopoietic marker) and CD45 (leukocyte antigen) (Figure 1(b)). These cells also expressed pluripotency markers of stem cells Oct4, Nanog, Sox2, and Rex1 as determined by RT-qPCR (Figure 1(c)). AF-MSCs were also confirmed for their multilineage differentiation potential towards osteogenic (Figure 1(d)), adipogenic, myogenic, and neurogenic lineages (data not shown).

### 3.2. Gene Expression during Osteogenic Differentiation of AF-MSCs

**Figure 2:**
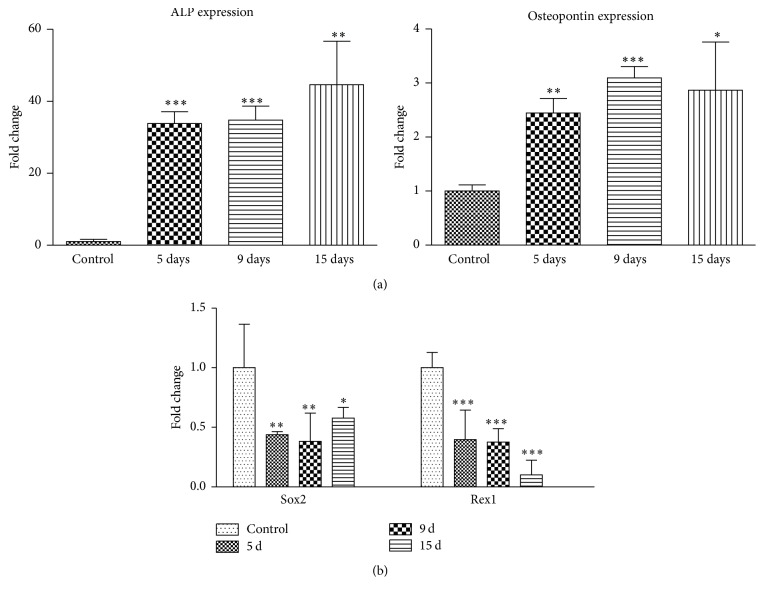
Gene expression analysis during osteogenic differentiation of AF-MSCs. (a) Relative* alkaline phosphatase (ALP)* and* osteopontin*, markers of osteogenic progenitors, gene expression. (b) Relative expression of genes, regulating pluripotency in stem cells:* Sox2* and* Rex1*. Gene expression was determined by RT-qPCR and data, normalized to GAPDH, are presented as *n*-fold change over control. Results are shown as mean ± SD (*n* = 3); ^*∗*^ *P* ≤ 0.05, ^*∗∗*^ *P* ≤ 0.001, and ^*∗∗∗*^ *P* ≤ 0.0001 indicate significant differences from control.

AF-MS cells cultured under osteogenic conditions for 15 days exhibited mineral calcium aggregates stained brightly red with Alizarin Red while control undifferentiated cells showed no staining (Figure 1(d)). Expression of two osteogenic differentiation genes markers,* alkaline phosphatase* and* osteopontin*, was determined using RT-qPCR (Figure 2(a)). We observed that relative expression of* alkaline phosphatase*, an early osteogenic differentiation marker, increased strongly (up to 50-fold) at the terminal osteogenic differentiation stage, that is, 15 days, compared to untreated AF-MSCs. On the other hand, we determined up to 3-fold increase in* osteopontin* gene expression, which is known as a late osteogenic marker. In addition to this, relative expression of stemness genes Sox2 and Rex1 was analyzed in AF-MSCs during induced osteogenic differentiation (Figure 2(b)). The results revealed Sox2 and Rex1 downregulation during differentiation relative to untreated control cells.

### 3.3. Expression of MicroRNA during Osteogenic Differentiation

**Figure 3:**
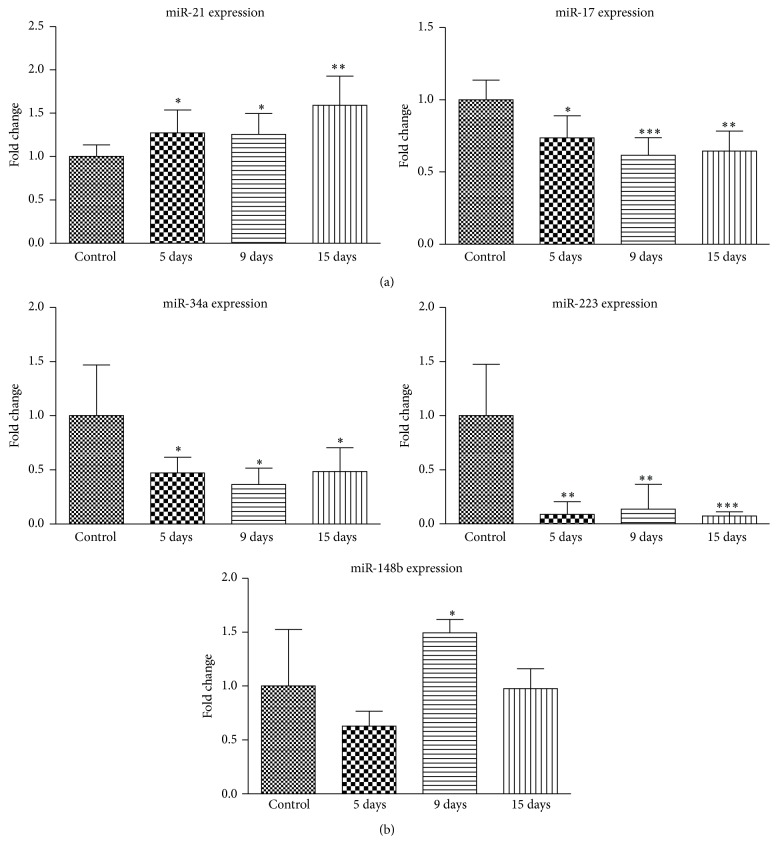
MicroRNA expression analysis during osteogenic differentiation of AF-MSCs. (a) Relative expression of miR-21 and miR-17 that are related to pluripotency maintenance in stem cells. (b) Relative expression of microRNAs 34a, 223, and 148b, which are upregulated or downregulated during osteogenic differentiation. MicroRNA expression was determined by RT-qPCR and data, normalized to RNU48, are presented as *n*-fold change over control. Results are shown as mean ± SD (*n* = 3); ^*∗*^ *P* ≤ 0.05, ^*∗∗*^ *P* ≤ 0.001, and ^*∗∗∗*^ *P* ≤ 0.0001 indicate significant differences from control.

MicroRNAs, small noncoding RNA molecules, regulate various genes related to development and differentiation of embryonic and other stem cells expression. Therefore we analyzed the levels of five different microRNAs, related to stem cells differentiation and renewal, after induced osteogenic differentiation of AF-MS cells. We chose two microRNAs which participate in pluripotency maintenance in stem cells, that is, miR-21 and miR-17 (Figure 3(a)). The relative expression, determined by RT-qPCR, showed that miR-21, which directly suppresses pluripotency marker Sox2, was upregulated during osteogenic differentiation. Levels of miR-17, which is upregulated in human pluripotent stem cells, were downregulated and decreased continuously during differentiation. In addition to this, we investigated relative levels of three microRNAs that regulate osteogenesis related genes (Figure 3(b)). The results showed downregulation of miR-34a, which inhibits osteoblast differentiation, as well as downregulation of miR-223 that mediates fate decisions between adipogenesis and osteogenesis. In addition, relative expression of miR-148b, osteogenesis promoting microRNA, was upregulated during osteogenic differentiation in our analyzed cells.

### 3.4. Epigenetic Changes during Osteogenic Differentiation of AF-MSCs

**Figure 4:**
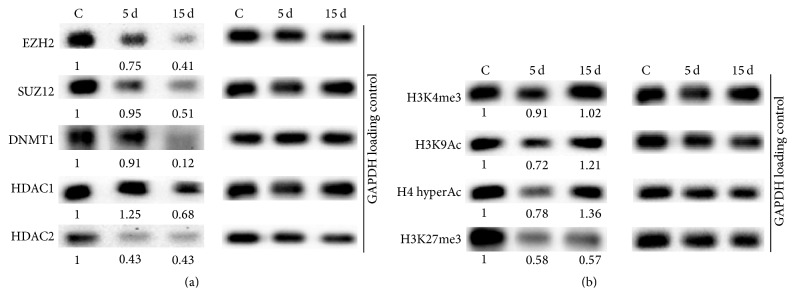
Epigenetic changes during osteogenic differentiation of AF-MSCs. Total proteins were isolated from untreated AF-MSCs (noted as C) and AF-MS cells at day 5 (noted as 5 d) and at day 15 (noted as 15 d) after induction of osteogenic differentiation. (a) Changes in the expression of PRC2 proteins, EZH2, SUZ12, DNMT1, HDAC1, and HDAC2. (b) Changes of histone modifications, H3K4me3, H3K9Ac, H4 hyperAc, and H3K27me3, levels. Numbers below blots show relative density of each band normalized to GAPDH loading control as measured using ImageJ software (NIH, USA). The data represent one of three independent experiments showing similar results.

In this study we explored epigenetic changes at proteins expression level and histone modifications at the initiation stage (day 5) and at the terminal stage of osteogenic differentiation (day 15) (Figure 4). Core members of Polycomb repressive complex 2 (PRC2), maintaining bivalent state of chromatin in stem cells, consist of histone H3 lysine 27 (H3K27) methyltransferase EZH2 and zinc-finger domain containing protein SUZ12. Our findings demonstrate that their expression is strongly downregulated at the terminal stage of osteogenic differentiation (Figure 4(a)). The expression levels of DNA and histones modifying enzymes DNA methyltransferase 1 (DNMT1) and histone deacetylases 1 (HDAC1) and 2 (HDAC2) also decreased during differentiation (Figure 4(a)). Further we examined the global levels of histone modifications considered as epigenetic markers. The data revealed minor changes in histone H3 lysine 4 trimethylation (H3K4me3) level and a slight upregulation of both acetylation of histone H3 lysine 9 (H3K9Ac) and histone H4 hyperacetylation (H4 hyperAc) as activating modifications. In correlation with decrease in PRC2 proteins levels, histone H3 lysine 27 trimethylation (H3K27me3), the chromatin compaction and inactivation mark, was downregulated after osteogenic induction in AF-MS cells (Figure 4(b)).

## 4. Discussion

Osteogenic differentiation and bone formation is a highly sophisticated process involving stimuli from surroundings and displaying changes in cell phenotype, transcriptional profile, and proteome as well as cellular communication and structures formation. These processes are tightly regulated by a network of signaling pathways and transcription factors. In addition, accumulating evidence indicates that epigenetic machinery by histone modifications and chromatin remodeling are also involved. In the present study we investigated phenotypical characteristics and gene and protein expression as well as modifications during osteogenic differentiation of amniotic fluid derived mesenchymal stem cells with the main focus on epigenetic changes.

Amniotic fluid derived mesenchymal stem cells expressed a comparable level of pluripotency maintenance markers in stem cells such as Oct4, Sox2, Nanog, and Rex1. AF-MSCs were defined as strongly expressing mesenchymal surface markers CD105 (endoglin) and CD90 (Thy-1, thymocyte antigen-1) and having no expression of CD34 (hematopoietic marker) and CD45 (leukocyte antigen). These characteristics correspond to the description of amniotic fluid derived mesenchymal stem cells isolated by other colleagues as reported previously [3, 4, 6].

We have chosen two widely used genes markers, that is, alkaline phosphatase (ALP) and osteopontin, as indicators of the presence of osteogenic differentiation. Upregulation of these genes and cell staining with Alizarin Red proved successful osteogenic differentiation [21–24].

Amniotic fluid derived mesenchymal stem cells are classified as multipotent and express pluripotency maintenance markers, transcription factors Oct4, Sox2, Nanog, and Rex1. It is known that during differentiation of stem cells the expression of these markers decreases as cells lose pluripotency and develop into specific cell line. All four markers of pluripotency form a convergent stem cell regulatory network, which controls the expression of key stem cell genes [35]. In the present study by using AF-MSC we determined that Sox2 and Rex1 were downregulated after induction of osteogenic differentiation. Sox2 along with Oct4 is at the top of the pluripotent regulatory network hierarchy as stated by Rodda et al. [36] and has been reported to promote proliferation by facilitating G1/S transition [35]. This is consistent with our data as AF-MSCs growth slows down during differentiation and Sox2 expression decreases. Moreover, Sox2 was identified as one of the Rex1 regulators together with other pluripotency markers [37] and lower levels of Sox2 correlate with lower expression of Rex1.

MicroRNAs (miR) are small and common regulators of gene expression and their expression alterations are related to various diseases or cancer development. Recently microRNAs were classified as epigenetic modifiers of gene expression in addition to other well-known epigenetic mechanisms. There are a number of microRNAs that are involved in stem cells self-renewal, maintenance of stemness, or differentiation. Due to this, expression of several microRNAs related to maintenance of pluripotency or modulation of osteogenesis in stem cells was analyzed in this study. Our results agree with reported studies showing that miR-21 directly suppresses Sox2 in human mesenchymal stem cells and promotes osteogenic differentiation [26] and levels of miR-17 that is specifically upregulated in human pluripotent stem cells [38] decreased after differentiation of AF-MSCs. An increasing amount of microRNAs has been demonstrated as regulators of different aspects of bone development or osteoblast differentiation. Some of them have been reported as inhibitors of osteogenic differentiation such as miR-34a [39] or miR-223 [40] in human stromal cells. As our results expose, the effect of these microRNAs is the same in human AF-MS cells as their levels significantly decreased after osteogenic differentiation induction. On the other hand, miR-148b is considered to enhance osteogenesis in adipose-derived stem cells [41] and we demonstrated slight but significant upregulation in miR-148b expression during osteogenic differentiation of AF-MSCs.

**Figure 5:**
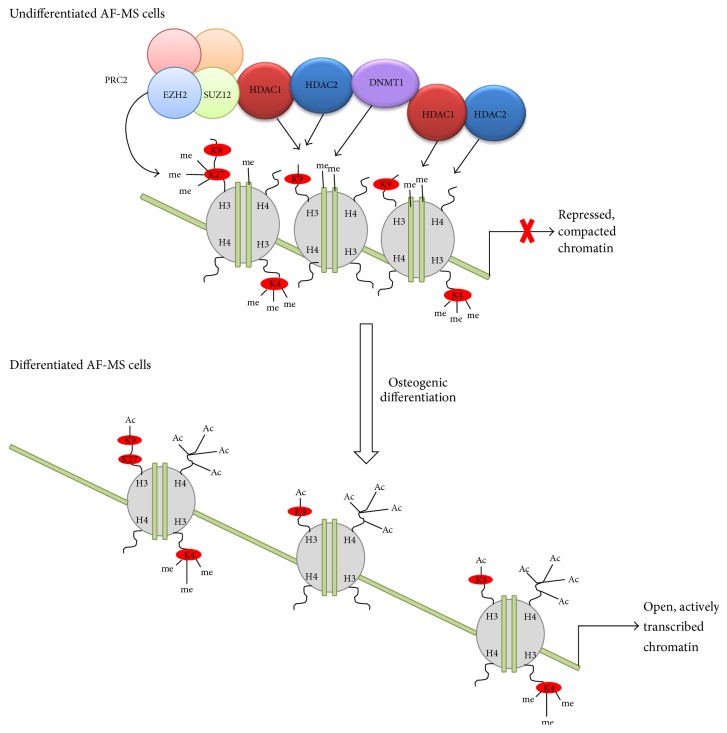
Schematic representation of chromatin remodeling conducted by epigenetic changes during osteogenic differentiation of AF-MSCs. Certain chromatin regions changeover from suppressed, silenced state to open, actively transcribed state via histone modifications (reduction of repressive methylation of H3K27 and upregulation of activating acetylation of H3K9 and H4) and related enzymes expression (decrease of PRC2 complex components EZH2 and SUZ12 as well as HDAC1/HDAC2 and DNMT1). Notably only components investigated in this study are shown as many more enzymes and modifications are involved in global epigenetic network.

Epigenetic changes we explored during osteogenic differentiation of AF-MSCs include analysis of chromatin modifying proteins and histone modifications. Our data revealed that expression of DNA methyltransferase 1 (DNMT1) that is responsible for maintenance of DNA methylation during replication decreased during osteogenic differentiation. This could be explained by loss of pluripotency and self-renewal properties because of cell commitment to differentiate when replication is no longer occurring. Our results are consistent with the report of Tsai and coauthors [42] where they showed that DNMT1 is regulated by pluripotency factors Oct4 and Nanog through direct binding to its promoter in adult mesenchymal stem cells and maintains self-renewal and undifferentiated state. In addition, during cell differentiation chromatin undergoes global changeover from repressed state when development-linked genes are inactive to open and actively transcribed state. Histones modifying enzymes are pivotal in mediating these changes and histone acetylation is one of the most abundant and dynamic histone modifications. It was reported that HDAC1 and HDAC2 inhibition enhances osteogenesis and blocks adipogenesis in mouse embryonic fibroblasts [33]. In agreement with this, our findings of downregulation of HDAC1 and HDAC2 as well as upregulation of acetylated forms of histones H3K9 and H4 show the importance of chromatin remodeling proceeding during AF-MSCs osteogenic differentiation. Another very significant player in epigenetic regulation of gene expression during differentiation in stem cells is PRC2 and its components, histone methyltransferase EZH2 and zinc-finger domain containing SUZ12. As our data indicate, EZH2 and SUZ12 expression was downregulated after induction of osteogenic differentiation of AF-MSCs and, as a consequence of this, trimethylation of H3K27 was reduced. Moreover, as stated in Wei et al. studies [32], EZH2-mediated decrease in H3K27me3 is controlled by CDK1-EZH2 signaling pathway and this is specific for the regulation of osteogenic differentiation of human MSCs through the modulation of EZH2-targeted osteogenic gene expression. Our findings demonstrate the importance of epigenetic changes (Figure 5) concomitant with the osteogenic differentiation of AF-MSCs.

## 5. Conclusions

In conclusion, osteogenic differentiation of human amniotic fluid derived mesenchymal stem cells is driven by epigenetic changes mediated by chromatin modifying enzymes, histone modifications, and specific microRNAs expression. All these factors together with transcription factors form a global integrated network that modulates the state of chromatin in maintaining self-renewal and pluripotency of stem cells as well as the differentiation processes. Our results extend the data of epigenetic processes during osteogenic differentiation and complement knowledge about mesenchymal stem cells derived from human amniotic fluid.

## Figures and Tables

**Table 1:** 

Gene	Primer sequence (5′-3′)	Product size (bp)
Oct4	Forward 5′-CGAGAAGGATGTGGTCCGAG	136
	Reverse 5′-CAGAGGAAAGGACACTGGTC	

Nanog	Forward 5′-AGATGCCTCACACGGAGACT	96
	Reverse 5′-GTTTGCCTTTGGGACTGGTG	

Sox2	Forward 5′-TGGACAGTTACGCGCACAT	215
	Reverse 5′-CGAGTAGGACATGCTGTAGGT	

Rex1	Forward 5′-GCCTTATGTGATGGCTATGTGT	96
	Reverse 5′-ACCCCTTATGACGCATTCTATGT	

Alkaline phosphatase (ALP)	Forward 5′-AGCCCTTCACTGCCATCCTGT	68
	Reverse 5′-ATTCTCTCGTTCACCGCCCAC	

Osteopontin	Forward 5′-GAAGTTTCGCAGACCTGACAT	91
	Reverse 5′-GTATGCACCATTCAACTCCTCG	

GAPDH	Forward 5′-AGTCCCTGCCACACTCAG	123
	Reverse 5′-TACTTTATTGATGGTACATGACAAGG	

## Abbreviations

AF-MSCs: Amniotic fluid derived mesenchymal stem cells
Oct4: Octamer-binding transcription factor 4
RT-qPCR: Quantitative reverse transcription polymerase chain reaction
Sox2: Sex determining region Y-box 2
Rex1: Reduced expression 1
EZH2: Enhancer of zeste homolog 2
SUZ12: Suppressor of zeste 12 protein homolog
FITC: Fluorescein isothiocyanate
PE: Phycoerythrin.

## References

[1] De Coppi P., Bartsch G., Siddiqui M. M. (2007). Isolation of amniotic stem cell lines with potential for therapy. *Nature Biotechnology*.

[2] Trohatou O., Anagnou N. P., Roubelakis M. G. (2013). Human amniotic fluid stem cells as an attractive tool for clinical applications. *Current Stem Cell Research and Therapy*.

[3] Prusa A.-R., Marton E., Rosner M., Bernaschek G., Hengstschläger M. (2003). Oct-4-expressing cells in human amniotic fluid: a new source for stem cell research?. *Human Reproduction*.

[4] Miranda-Sayago J. M., Fernández-Arcas N., Benito C., Reyes-Engel A., Carrera J., Alonso A. (2011). Lifespan of human amniotic fluid-derived multipotent mesenchymal stromal cells. *Cytotherapy*.

[5] Tsai M.-S., Lee J.-L., Chang Y.-J., Hwang S.-M. (2004). Isolation of human multipotent mesenchymal stem cells from second-trimester amniotic fluid using a novel two-stage culture protocol. *Human Reproduction*.

[6] Savickiene J., Treigyte G., Baronaite S. (2015). Human amniotic fluid mesenchymal stem cells from second- and third-trimester amniocentesis: differentiation potential, molecular signature, and proteome analysis. *Stem Cells International*.

[7] Roubelakis M. G., Pappa K. I., Bitsika V. (2007). Molecular and proteomic characterization of human mesenchymal stem cells derived from amniotic fluid: comparison to bone marrow mesenchymal stem cells. *Stem Cells and Development*.

[8] Pesce M., Schöler H. R. (2001). Oct-4: gatekeeper in the beginnings of mammalian development. *STEM CELLS*.

[9] Karlmark K. R., Freilinger A., Marton E., Rosner M., Lubec G., Hengstschläger M. (2005). Activation of ectopic Oct-4 and Rex-1 promoters in human amniotic fluid cells. *International Journal of Molecular Medicine*.

[10] Bossolasco P., Montemurro T., Cova L. (2006). Molecular and phenotypic characterization of human amniotic fluid cells and their differentiation potential. *Cell Research*.

[11] In 't Anker P. S., Scherjon S. A., Kleijburg-van der Keur C. (2003). Amniotic fluid as a novel source of mesenchymal stem cells for therapeutic transplantation. *Blood*.

[12] Joo S., Ko I. K., Atala A., Yoo J. J., Lee S. J. (2012). Amniotic fluid-derived stem cells in regenerative medicine research. *Archives of Pharmacal Research*.

[13] Kaviani A., Perry T. E., Dzakovic A., Jennings R. W., Ziegler M. M., Fauza D. O. (2001). The amniotic fluid as a source of cells for fetal tissue engineering. *Journal of Pediatric Surgery*.

[14] Kassem M., Kristiansen M., Abdallah B. M. (2004). Mesenchymal stem cells: cell biology and potential use in therapy. *Basic and Clinical Pharmacology and Toxicology*.

[15] Li C., Zhou J., Shi G. (2009). Pluripotency can be rapidly and efficiently induced in human amniotic fluid-derived cells. *Human Molecular Genetics*.

[16] Drews K., Matz P., Adjaye J. (2015). Generation of iPSC lines from primary human amniotic fluid cells. *Stem Cell Research*.

[17] Ye L., Chang J. C., Lin C., Sun X., Yu J., Kan Y. W. (2009). Induced pluripotent stem cells offer new approach to therapy in thalassemia and sickle cell anemia and option in prenatal diagnosis in genetic diseases. *Proceedings of the National Academy of Sciences of the United States of America*.

[18] Qin M., Chen R., Li H. (2016). Direct reprogramming of human amniotic fluid stem cells by OCT4 and application in repairing of cerebral ischemia damage. *International Journal of Biological Sciences*.

[19] Mauro A., Turriani M., Ioannoni A. (2010). Isolation, characterization, and in vitro differentiation of ovine amniotic stem cells. *Veterinary Research Communications*.

[20] Liu Z. S., Xu Y. F., Feng S. W. (2009). Baculovirus-transduced mouse amniotic fluid-derived stem cells maintain differentiation potential. *Annals of Hematology*.

[21] de Lara Janz F., Favero G. M., Bohatch M. S., Aguiar Debes A., Bydlowski S. P. (2014). Simvastatin induces osteogenic differentiation in human amniotic fluid mesenchymal stem cells (AFMSC). *Fundamental and Clinical Pharmacology*.

[22] Liu M., Li Y., Yang S.-T. (2014). Curculigoside improves osteogenesis of human amniotic fluid-derived stem cells. *Stem Cells and Development*.

[23] Liu M., Li Y., Yang S. T. (2014). Effects of naringin on the proliferation and osteogenic differentiation of human amniotic fluid-derived stem cells. *Journal of Tissue Engineering and Regenerative Medicine*.

[24] Zavatti M., Resca E., Bertoni L. (2013). Ferutinin promotes proliferation and osteoblastic differentiation in human amniotic fluid and dental pulp stem cells. *Life Sciences*.

[25] De Rosa A., Tirino V., Paino F. (2011). Amniotic fluid-derived mesenchymal stem cells lead to bone differentiation when cocultured with dental pulp stem cells. *Tissue Engineering—Part A*.

[26] Trohatou O., Zagoura D., Bitsika V. (2014). Sox2 suppression by miR-21 governs human mesenchymal stem cell properties. *Stem Cells Translational Medicine*.

[27] Berardinelli P., Valbonetti L., Muttini A. (2013). Role of amniotic fluid mesenchymal cells engineered on MgHA/collagen-based scaffold allotransplanted on an experimental animal study of sinus augmentation. *Clinical Oral Investigations*.

[28] Maraldi T., Riccio M., Resca E. (2011). Human amniotic fluid stem cells seeded in fibroin scaffold produce in vivo mineralized matrix. *Tissue Engineering—Part A*.

[29] Meissner A., Mikkelsen T. S., Gu H. (2008). Genome-scale DNA methylation maps of pluripotent and differentiated cells. *Nature*.

[30] Fouse S. D., Shen Y., Pellegrini M. (2008). Promoter CpG methylation contributes to ES cell gene regulation in parallel with Oct4/Nanog, PcG complex, and histone H3 K4/K27 trimethylation. *Cell Stem Cell*.

[31] Barrand S., Collas P. (2010). Chromatin states of core pluripotency-associated genes in pluripotent, multipotent and differentiated cells. *Biochemical and Biophysical Research Communications*.

[32] Wei Y., Chen Y.-H., Li L.-Y. (2011). CDK1-dependent phosphorylation of EZH2 suppresses methylation of H3K27 and promotes osteogenic differentiation of human mesenchymal stem cells. *Nature Cell Biology*.

[33] Haberland M., Carrer M., Mokalled M. H., Montgomery R. L., Olson E. N. (2010). Redundant control of adipogenesis by histone deacetylases 1 and 2. *Journal of Biological Chemistry*.

[34] Hemming S., Cakouros D., Isenmann S. (2014). EZH2 and KDM6A act as an epigenetic switch to regulate mesenchymal stem cell lineage specification. *Stem Cells*.

[35] Chen Y., Shi L., Zhang L. (2008). The molecular mechanism governing the oncogenic potential of SOX2 in breast cancer. *The Journal of Biological Chemistry*.

[36] Rodda D. J., Chew J.-L., Lim L.-H. (2005). Transcriptional regulation of Nanog by OCT4 and SOX2. *The Journal of Biological Chemistry*.

[37] Shi W., Wang H., Pan G., Geng Y., Guo Y., Pei D. (2006). Regulation of the pluripotency marker Rex-1 by Nanog and Sox2. *Journal of Biological Chemistry*.

[38] Leonardo T. R., Schultheisz H. L., Loring J. F., Laurent L. C. (2012). The functions of microRNAs in pluripotency and reprogramming. *Nature Cell Biology*.

[39] Chen L., Holmstrøm K., Qiu W. (2014). MicroRNA-34a inhibits osteoblast differentiation and in vivo bone formation of human stromal stem cells. *Stem Cells*.

[40] Guan X., Gao Y., Zhou J. (2015). miR-223 regulates adipogenic and osteogenic differentiation of mesenchymal stem cells through a C/EBPs/miR-223/FGFR2 regulatory feedback loop. *STEM CELLS*.

[41] Liao Y.-H., Chang Y.-H., Sung L.-Y. (2014). Osteogenic differentiation of adipose-derived stem cells and calvarial defect repair using baculovirus-mediated co-expression of BMP-2 and miR-148b. *Biomaterials*.

[42] Tsai C.-C., Su P.-F., Huang Y.-F., Yew T.-L., Hung S.-C. (2012). Oct4 and nanog directly regulate dnmt1 to maintain self-renewal and undifferentiated state in mesenchymal stem cells. *Molecular Cell*.

